# Acoustic Imaging with Metamaterial Luneburg Lenses

**DOI:** 10.1038/s41598-018-34581-7

**Published:** 2018-11-01

**Authors:** Yangbo Xie, Yangyang Fu, Zhetao Jia, Junfei Li, Chen Shen, Yadong Xu, Huanyang Chen, Steven A. Cummer

**Affiliations:** 1 Department of Electrical and Computer Engineering, Duke University, Durham, North Carolina 27708 USA; 2 Institute of Electromagnetics and Acoustics and Department of Electronic Science, Xiamen University, Xiamen, 361005 China; 3 College of Physics, Optoelectronics and Energy, Soochow University, No.1 Shizi Street, Suzhou, 215006 China; 4 College of Science, Nanjing University of Aeronautics and Astronautics, Nanjing, 211106 China

## Abstract

The Luneburg lens is a spherically symmetrical gradient refractive index (GRIN) device with unique imaging properties. Its wide field-of-view (FoV) and minimal aberration have lead it to be successfully applied in microwave antennas. However, only limited realizations have been demonstrated in acoustics. Previously proposed acoustic Luneburg lenses are mostly limited to inherently two-dimensional designs at frequencies from 1 kHz to 7 kHz. In this paper, we apply a new design method for scalable and self-supporting metamaterials to demonstrate Luneburg lenses for airborne sound and ultrasonic waves. Two Luneburg lenses are fabricated: a 2.5D ultrasonic version for 40 kHz and a 3D version for 8 kHz sound. Imaging performance of the ultrasonic version is experimentally demonstrated.

## Introduction

The Luneberg lens^1^ is a representative GRIN lens that offers a variety of unique wave controlling properties: first, a Luneberg lens bends a plane wave to a focal spot on the opposite spherical surface of the lens, which is a very attractive feature in imaging since the lens maps the direction information directly to the spatial locations of the focuses. Secondly, a Luneberg lens has spherical symmetry (or cylindrical symmetry for the two-dimensional case), which brings in advantages of minimal spherical aberration and a wide FoV. In principle, even a full solid-angular coverage of FoV can be achieved if the sensor is transparent to the incoming wave. Last but not least, a Luneburg lens shares many advantages typical for GRIN devices^2–4^: refractive index profiles are non-singular, non-resonant structures are needed, and the operating bandwidths are usually larger compared to the diffractive devices^5–8^ that achieve similar functionalities.

While a variety of Luneburg lenses for radio frequency electromagnetic waves^9^, optical waves^10^, surface electromagnetic waves^11^, surface plasmon polaritons (SPPs)^12^, and elastic waves^13^ have been realized with the help of metamaterials in the recent past, acoustic Luneburg lenses have been less explored. Previous realizations of acoustic Luneburg lenses are limited to inherently two-dimensional designs and audible frequency range (1 kHz to 7 kHz)^14,15^. Designing three-dimensional acoustic Luneburg lens requires designing and fabrication of structures with complex three-dimensional geometries with overhanging structures at subwavelength precision. Therefore, it remains challenging to design a true three-dimensional acoustic Luneburg lens, particularly for the airborne ultrasonic frequency range.

In this work, we design and fabricate two GRIN Luneburg lenses: a 2.5D version for 40 kHz ultrasound and a 3D version for 8 kHz sound. The designs are based on a series of 3D cross-shaped metamaterial structures that can be stacked layer-by-layer to form a stable lattice^16^. We also demonstrate here the experimental characterization of the performance of the 2.5D ultrasonic Luneburg lens. Two imaging experiments were implemented to demonstrate its functionalities in imaging: finding the direction of a single source and resolving multiple sources. The experimental platform is described, and the measurement results are presented and compared with the simulation results. The proposed ultrasonic Luneburg lens can be potentially useful for enhancing the sensing performance of existing pulse-echo-based airborne ultrasonic sensors and imaging systems (e.g. shaping the radiation pattern, extending the sensing range), and the audible version may be used for improving the radiation pattern for speaker systems. Moreover, we expect many other three-dimensional GRIN devices can be conveniently realized with similar methods for better control of airborne acoustic waves.

## Results

### Building blocks for the lens: 3D scalable and self-supporting metamaterials

Common solid materials such as metal and plastics have close-to-infinite impedance contrast with air for acoustic waves. However, combining high impedance material with air will form composites with finite impedances. By varying the filling ratio of the high impedance material, a range of above-unity refractive index can be achieved. Previous two-dimensional designs based on such a filling-fraction composite have been successfully demonstrated^17^.

**Figure 1:**
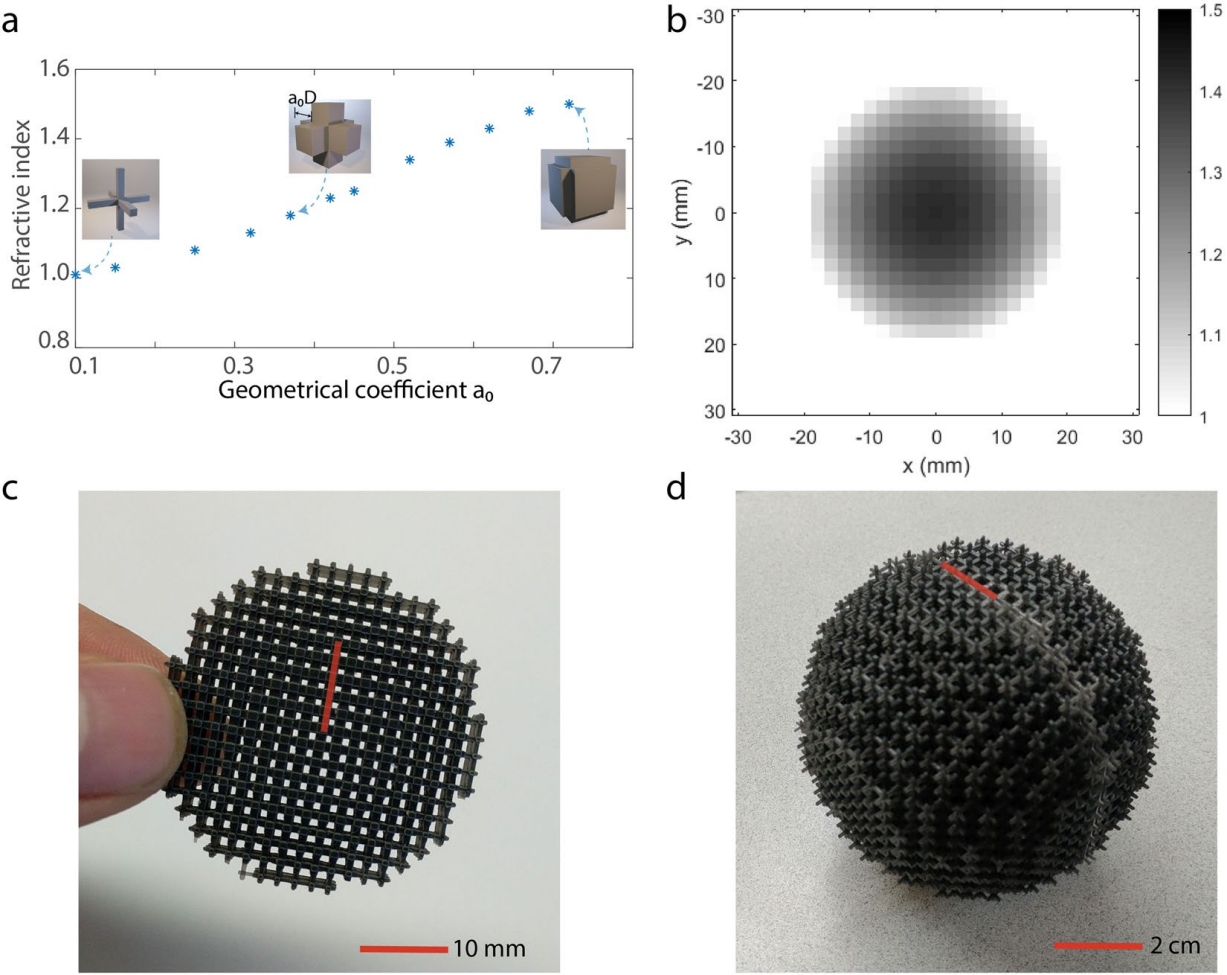
Designing Luneburg lenses. (**a**) The building blocks of the Luneburg lens: 3D-crosses with varying geometric coefficient a_0_ lead to a range of refractive index. In the inset, the dimension of a_0_D is marked (D is the length of the unit cell, which is 2 mm for the ultrasonic version). (**b**) The refractive index profile of a 40 kHz Luneburg lens. (**c**) A 2.5D sample design for 40 kHz airborne ultrasound. There are three layers of the 3D-cross unit cells along the out-of-plane dimension. (**d**) A full 3D sample design for 8 kHz airborne sound.

To realize a three-dimensional GRIN device, there is an additional design constrain that the structures need to be self-supporting and be mechanically stable for overlaying layers. As a result, many previously reported 2D designs cannot be directly applied here. To tackle this challenge, we designed a series of 3D-cross-shaped metamaterial structures as our building block. Each 3D-cross with its surrounding air acts as a subwavelength cubic unit cell and a 3D array of such unit cells form a structurally stable cubic lattice. The family of these metamaterial unit cells is illustrated in Fig. 1a. By varying the dimensions of the 3D-cross through a geometrical coefficient a_0_ as shown in the inset in Fig. 1a, a refractive index ranging from 1 to 1.5 can be achieved. Such design has several advantages: (1) the lattice structure can stably build up in a layer-by-layer fashion to form a 3D spatially inhomogeneous device; (2) the cubic unit cells are subwavelength and have isotropic effective wave properties; (3) the design has broader bandwidth than resonant metamaterial structures^18–22^ and the refractive index contrast is relatively constant over about 25% of the central frequency; (4) the structures are directly 3D printable with commercially available printers for frequencies up to 40 kHz. When the geometric coefficient a_0_ is 0.75, the refractive index of a building block is about 1.772, which is about the upper limit of a 3D-cross-shaped metamaterial design that can be practically realized. The impedance of a building block also increases with the refractive index. However, the spatially gradient transitioning of the refractive index of a GRIN device minimizes the influence of the non-unity impedance^16^.

Manual generation of the needed 3D structures can be an extremely time-consuming task. Here we used an automated CAD tool called GRIPP^14^ to map the refractive index profile to the realistic 3D-cross structures and automatically create the structural layout for straightforward 3D printing. GRIPP is a universal design tool for achieving a large range of GRIN devices: it takes the input of the spatial distribution of the refractive index and the preferred unit cell library, then automatically generates a 3D printable structure of the GRIN device. The technical details of GRIPP are described in ref.^16^.

### Designing 2.5D and 3D Luneburg lenses

To demonstrate our design method, here we present two acoustic GRIN lenses designed with the above-mentioned unit cell structures and CAD tool. Both GRIN lenses are Luneburg lenses with refractive index that follows the formula $n=\sqrt{2-{(\frac{r}{R})}^{2}}$. The target index distribution for an ultrasonic 2.5D Luneburg lens is shown in Fig. 1b. Its spatial inhomogeneity is in its two-dimensional plane and the plane is extruded uniformly by three layers of unit cells in the third dimension. The lens is designed for 40 kHz airborne ultrasound, which is widely applied for distance ranging and obstacle detection. The unit cell size of this design is 2 mm, which is about 23.3% of the wavelength. The smallest feature (e.g., a hole around the center) of the design is about 760 μm. This ultrasonic sample is fabricated with a PolyJet 3D printer and is shown in Fig. 1c. The second sample is a 3D Luneburg lens designed for 8 kHz sound, shown in Fig. 1d. The unit cell size of this design is 5 mm, which is about 11.7% of the wavelength. Due to the larger volume of this design, PolyJet printing would be unnecessarily costly; Instead, the 3D sample is fabricated with a commercially available stereolithography (SLA) printer. Two hemispheres were independently printed and jointed with a Ultraviolet (UV) bonding process. More details on the fabrication can be found in the Methods section.

### Experimental characterization of the 2.5D ultrasound Luneburg lens

**Figure 2:**
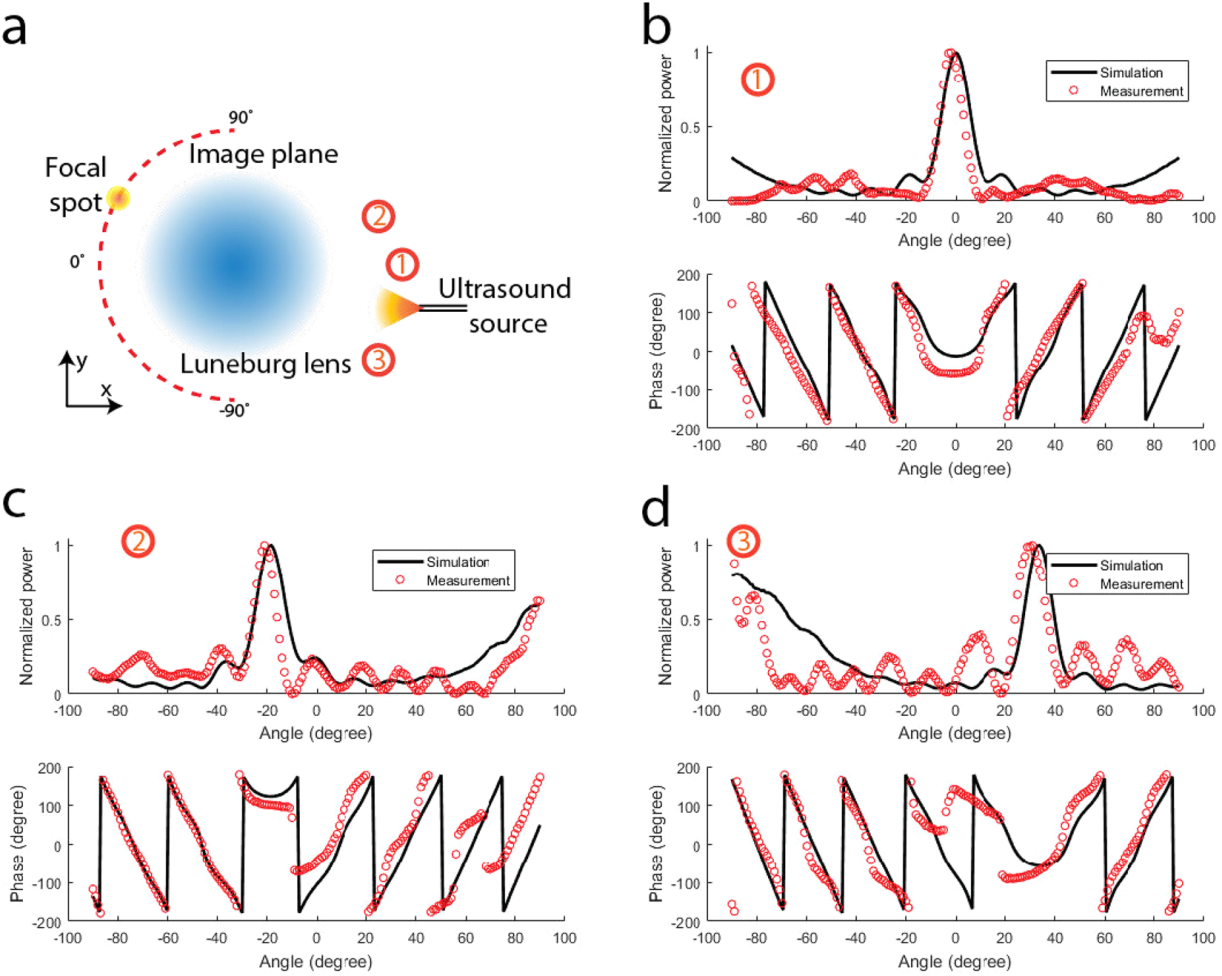
The setup and the measured results of the 2D ultrasonic source direction finding experiments. (**a**) The experimental setup. A single ultrasonic source is placed on the right-hand side of the lens. The pressure along the semi-circle focal curve with 28 mm radius on the left-hand side of the lens is measured. (**b**) The comparison between the measured and the simulated pressure amplitude and phase along the focal curve for a single ultrasonic source in coordinate (35 mm, 0 mm). (**c**) The comparison between the measured and the simulated pressure amplitude and phase along the focal curve for a single ultrasonic source in coordinate (30 mm, 10 mm). (**d**) The comparison between the measured and the simulated pressure amplitude and phase along the focal curve for a single ultrasonic source in coordinate (30 mm, −20 mm).

In this section, we present the experiment characterization of the 40 kHz Luneburg lens. We first demonstrate here that a Luneburg lens can be used for direction finding of the sources, without the need of computational beamforming. As shown in Fig. 2a, an ultrasonic source is placed on the right side of the lens; on the left side is the semi-circular focal surface (red dashed curve). A Luneburg lens is known for its ability to focus an incident plane wave to a focal point on its surface. For a point source placed infinitely far away from the lens, the incoming beam is close to plane wave and the focal spot will be formed on the edge of the Luneburg lens; for a source placed at finite distance away from the lens (as in the case of our experiment), the focal plane will be finite distance away from the edge of the lens.

Ideally, an array of sensors will be deployed on the curved focal surface, so the measured amplitude distribution will inform the direction of the source. For the convenience of the experiment, we scanned the focal surface with a single receiver. A variety of source locations were tested and here we selected three representative locations where their images form at about 0, -20 and 40 degrees respectively. The comparison between the measurements and the simulated results were shown in Fig. 2b–d. The amplitude distribution along the focal curve clearly shows peaks corresponding to the image of the sources. The measured amplitude and phase have excellent agreement with those extracted from the simulations. The location of the image along the focal curve indicates the source location. As a result, the source direction and the image location have an isomorphic mapping, thus no computation such as beamforming is required to solve the inverse problem of finding the direction of the source.

**Figure 3:**
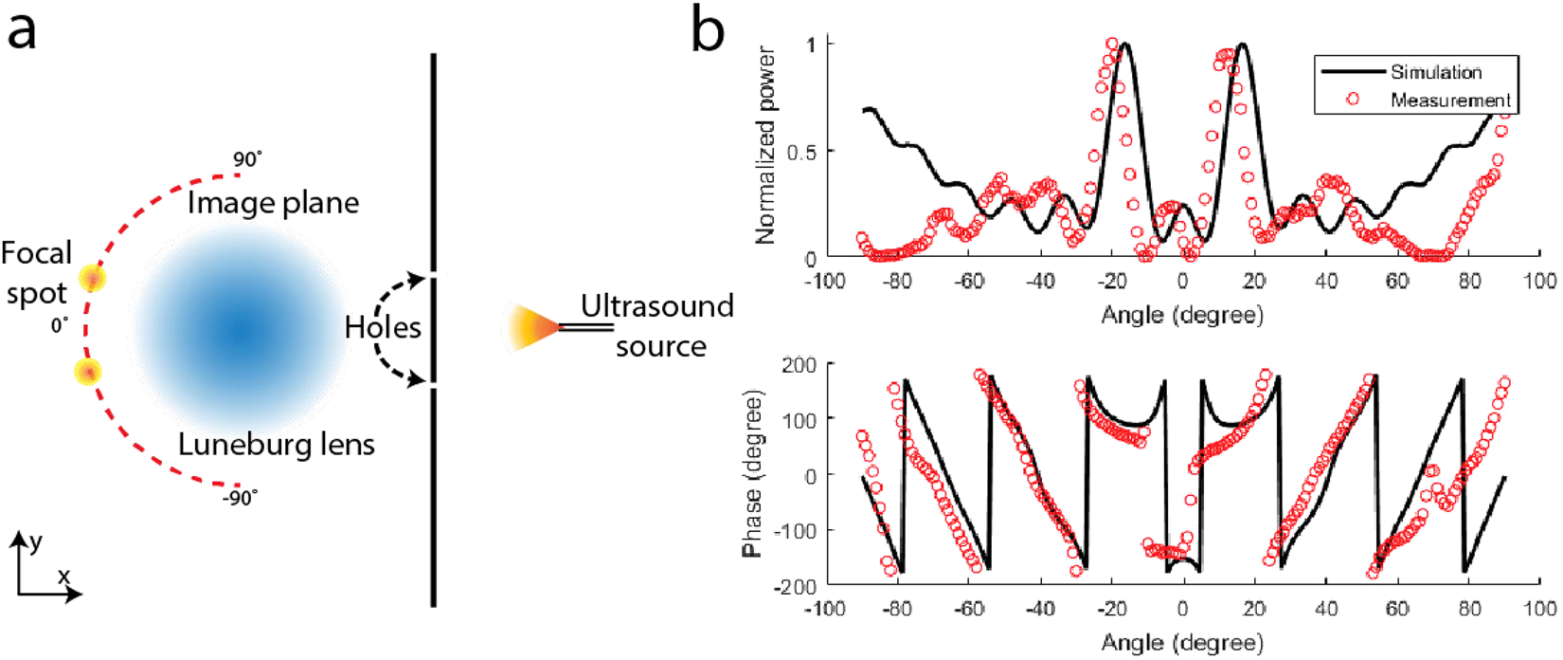
The setup and the measured results of resolving two ultrasonic sources. (**a**) The experimental setup. A single ultrasonic source is placed behind a sound hard wall with two 1 mm diameter holes at coordinate (40 mm, −10 mm) and (40 mm, 10 mm) respectively. The pressure along the semi-circle focal curve on the left-hand side of the lens is measured. (**b**) The comparison between the measured and the simulated pressure amplitude and phase along the focal curve.

To verify that the Luneburg lens can resolve multiple sources, we designed a second experiment to demonstrate the imaging capability of the Luneburg lens. A sound-hard plate with two circular holes (1 mm diameter) was placed 40 mm away from the center of the lens. An ultrasonic source illuminating the plate forms a pair of sources, as shown in Fig. 3a. When we measured the sound field on the curved focal surface, two sources were clearly resolved, as shown in Fig. 3b. The measurement is in excellent agreement with the prediction from simulation, with the two peaks slightly displaced due to the imperfect alignment of the holes. This simple imaging experiment demonstrate that such lens can be used not only for direction finding, but also can resolve multiple acoustic sources directly on the focal curve.

## Discussion and Conclusions

A number of potential applications are enabled by ultrasound lenses such as the Luneburg lens described above. By shaping the airborne acoustic wavefront, acoustic radiation can be collimated to allow longer propagation distance or be concentrated to an energy hotspot. The design and demonstration of airborne ultrasound lens may impact a variety of sensing applications that utilizes ultrasonic waves (mostly 30 kHz to 200 kHz) that propagate in air. For example, smart vehicles typically equip ultrasonic sonars to sense the environment to assist self-parking or auto pilot. In addition, particle levitation^23^ and wireless power transfer for cable-free transmission of energy are emerging applications of airborne ultrasound.

Conventional acoustic imaging methods, such as beamforming or near-field acoustic holography (NAH), require a non-trivial amount of processing to deliver images. While the computational power is not always the bottleneck in the system design, reducing computational complexity is still sought-after for a variety of sensing and imaging systems used in resource-limited environments. Lens-based acoustic imaging can be an attractive option for addressing such challenge. First, like focal optical imaging, a lens-based imaging system can form the object-image mapping directly through the lensing effects without the need of computationally intensive parallel channel processing (as in the case of phased-array beamforming imaging method). In the future, flexible pressure amplitude sensor plane with visual output (e.g. LED array) can be used to directly form a ‘what-you-see-is-what-you-measure’ image on the sensor plane. The second advantage is the reduced hardware cost and the increased system robustness, since the field transformation is implemented with a passive lens rather than an array of active sensors (together with corresponding delaying/readout circuitry). The third advantage is the improved image frame rate: since the sensor only needs to measure the instantaneous amplitude distribution rather than the full acoustic waveform, the frame-rate is no longer restricted by the duration of the probing pulse.

In summary, we demonstrated the design and performances of Luneburg lenses for airborne acoustic waves. We first designed two Luneburg lenses based on scalable and self-supporting 3D-cross structures and generated the whole 3D design with a CAD tool. Second, we showed that additive manufacturing can be leveraged to conveniently realize these gradient index devices: PolyJet for airborne ultrasonic frequency range (40 kHz) and SLA for lower frequency (8 kHz). Third, we experimentally characterized the 2.5D Luneburg lens designed for 40 kHz airborne ultrasound with two experiments: the direction finding of a single source and the resolving of two sources. Excellent agreement with simulation results were obtained.

The method can be straightforwardly extended to design a large range of spatially inhomogeneous GRIN devices, such as transformation acoustics-based wave controlling devices^24,25^. The GRIN devices based on the proposed method may impact applications in areas such as audio engineering, ultrasound detectors and sensors.

## Methods

### Numerical simulation

All the FEM simulation presented in paper are extracted from a commercial finite-element method-based software, COMSOL Multiphysics 5.3 (Pressure Acoustics module). The Luneburg lenses in the simulation have effective refractive index and unity impedance.

### Sample fabrication

The 40 kHz two-dimensional Luneburg lens was fabricated with a Stratasys J750 PolyJet printer. The printed sample was embedded in a jelly-like support material and a high-pressure water splashing machine was utilized to remove the support material. The 8 kHz three-dimensional Luneburg lens was fabricated with a Form 2 SLA printer. The spherical design was divided into two hemispheres to eliminate the necessity of extensive supporting structures. The two independently printed hemispheres were jointed with a UV bonding process.

### Imaging experiment setup

**Figure 4:**
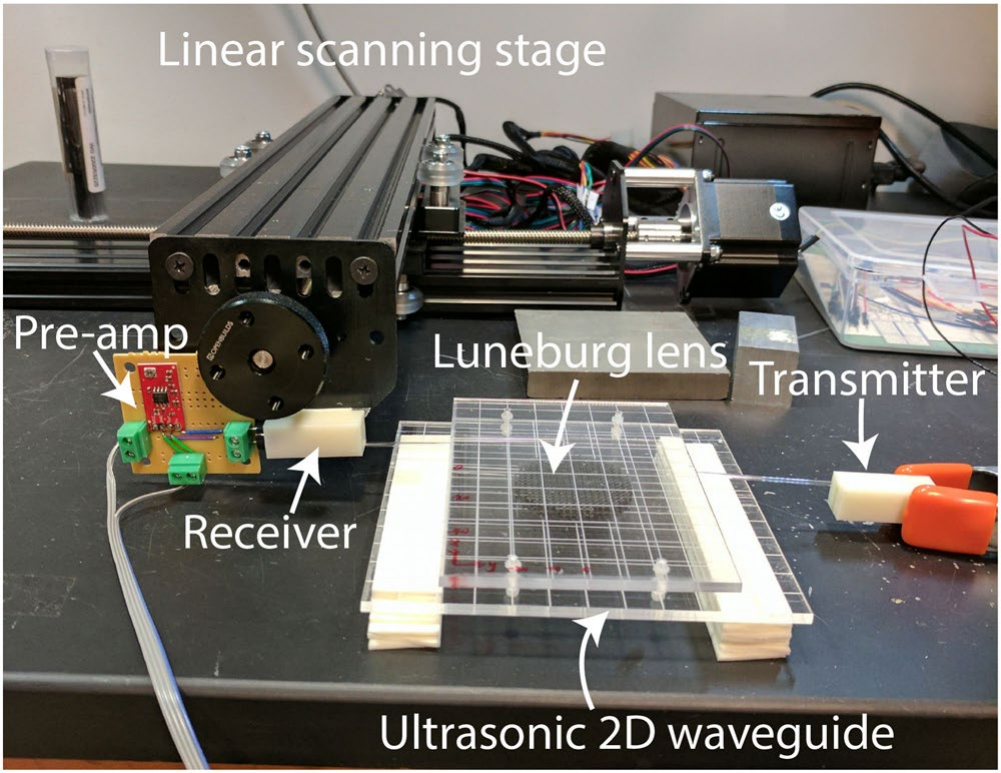
The experimental setup of the ultrasonic imaging experiment.

The measurement platform for the lensing experiment descried in the previous section is essentially a scaled-down version of the 2D scanning stage we have used for kilohertz field-mapping experiments^6,26^. As shown in Fig. 4, an ultrasonic waveguide was fabricated with laser-cut acrylic plates to confine the transmitted ultrasonic wave in a quasi-two-dimensional space. A pair of Murata ultrasound transducers (part number MA40S4S and MA40S4R) were used as the transmitter and the receiver. Two 3D printed tapered waveguiding adapters with glass tubes (about 1 mm diameter) were used to guide the ultrasonic wave from the transmitter into the waveguide, and guide the received wave from the waveguide to the receiver transducer. A LM358-based operational amplifier was used as the pre-amplification system. The pre-amplified signal was transmitted then to and be digitized by the NI PCI-6251 data-acquisition system. A two-dimensional linear stage was programmed to scan the field along the pre-defined trajectory along the focal curve.

## Electronic supplementary material

Supplementary Information
