# Supplementary material for: Acoustic Imaging with Metamaterial Luneburg Lenses
Source: Sci Rep. 2018 Nov 1;8:16188. doi: 10.1038/s41598-018-34581-7 (PMC6212425; doi:10.1038/s41598-018-34581-7)
Supplement: Supplementary file 1 — Supplementary Information [file 41598_2018_34581_MOESM1_ESM.pdf]

Supplementary Information

Acoustic Imaging with Metamaterial Luneburg Lenses

Yangbo Xie[1]*, Yangyang Fu[1,2,3]*, Zhetao Jia[1], Junfei Li[1], Chen Shen[1], Yadong Xu[3], Huanyang Chen[2] and Steven A. Cummer[1]

1. *Department of Electrical and Computer Engineering, Duke University, Durham, North Carolina 27708, USA*
2. *Institute of Electromagnetics and Acoustics and Department of Electronic Science, Xiamen University, Xiamen 361005, China.*
3. *College of Physics, Optoelectronics and Energy, Soochow University, No.1 Shizi Street, Suzhou 215006, China.*

* These authors contributed equally to this work

[†]Corresponding authors:
cummer@ee.duke.edu
kenyon@xmu.edu.cn

Supplementary Methods 1: The retrieval method for obtaining the effective parameters of the unit cell

The effective refractive index of a 3D-cross-shaped unit cell is calculated with a standard transmission-reflection-based retrieval method[1]: one waveguide that has the same subwavelength cross-section as the unit cell is excited and only fundamental mode can propagate in the waveguide. The unit cell to be retrieved is placed in the center of the waveguide (the black frame of the unit cell only indicates the dimension of the unit cell). The structure (colored in blue) is composed of ABS plastics. S11 and S21 are retrieved with the simulation and the effective refractive index and effective impedance of the unit cell can then be calculated using the formula in ref. 1.

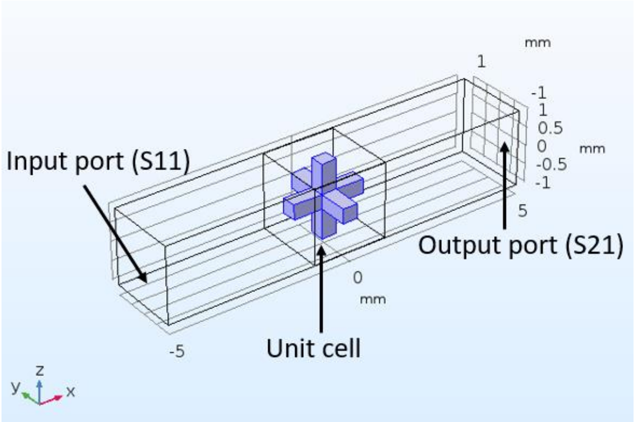

Fig. S1 | The model for retrieving the effective refractive index of a 3D-cross-shaped unit cell.

Supplementary Information 1: Modeling the loss of the 3D printed acoustic Luneburg lens

We model the loss as imaginary part in the refractive index. The imaginary part is an empirical value obtained by comparing the measured the pressure amplitude response with simulated responses where the Luneburg lens has various lossy coefficients. In the following two cases, we multiple the refractive index of the Luneburg lens with the following lossy coefficient: $n_0 = 1$, $n_0 = 1 - 0.02i$, $n_0 = 1 - 0.04i$ and $n_0 = 1 - 0.06i$. The result shows that $n_0 = 1 - 0.02i$ has the optimal overall match between simulation and measurements. Therefore, we use the following formula to model the effective lossy refractive index of the Luneburg lens in our simulations: $n = (1 - 0.02i)\sqrt{2 - \left(\frac{r}{R}\right)^2}$.

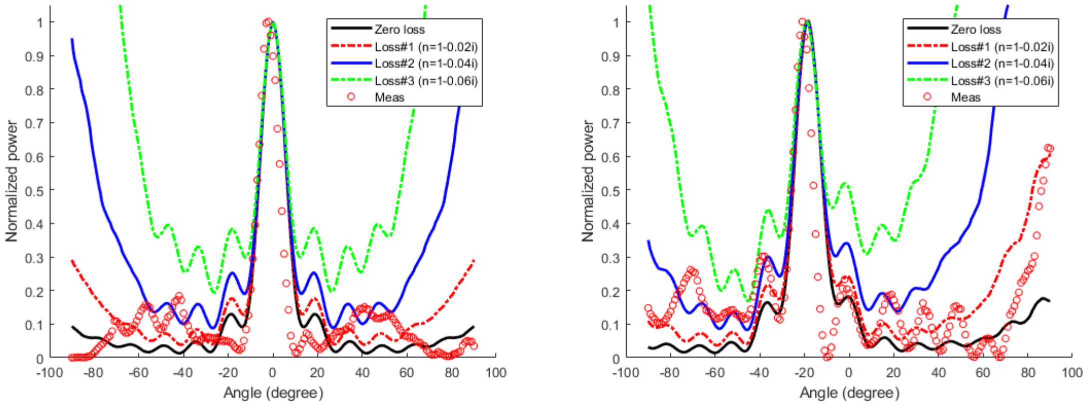

Fig. S2 | Left panel: the comparison between the measured and the simulated pressure amplitude distributions along the focal curve for a single ultrasonic source in coordinate (35 mm, 0 mm). The simulation uses four different lossy coefficients and n=1-0.02i has the best overall match. Right panel: the comparison between the measured and the simulated pressure amplitude distributions along the focal curve for a single ultrasonic source in coordinate (35 mm, 10 mm). The simulation uses four different lossy coefficients and n=1-0.02i again has the best overall match.

Supplementary References

[1] Fokin, V., Ambati, M., Sun, C. & Zhang, X. Method for retrieving effective properties of locally resonant acoustic metamaterials. *Phys. Rev. B* 76, 144302 (2007).
